# Optimizing Sample Size to Assess the Genetic Diversity in Common Vetch (*Vicia sativa* L.) Populations Using Start Codon Targeted (SCoT) Markers

**DOI:** 10.3390/molecules22040567

**Published:** 2017-03-31

**Authors:** Xutian Chai, Rui Dong, Wenxian Liu, Yanrong Wang, Zhipeng Liu

**Affiliations:** The State Key Laboratory of Grassland Agro-Ecosystems, College of Pastoral Agriculture Science and Technology, Lanzhou University, Lanzhou 730020, China; chaixt15@lzu.edu.cn (X.C.); dongr13@lzu.edu.cn (R.D.); liuwx@lzu.edu.cn (W.X.); yrwang@lzu.edu.cn (Y.W.)

**Keywords:** *Vicia sativa*, SCoT marker, sampling size, genetic diversity

## Abstract

Common vetch (*Vicia sativa* subsp. *sativa* L.) is a self-pollinating annual forage legume with worldwide importance. Here, we investigate the optimal number of individuals that may represent the genetic diversity of a single population, using Start Codon Targeted (SCoT) markers. Two cultivated varieties and two wild accessions were evaluated using five SCoT primers, also testing different sampling sizes: 1, 2, 3, 5, 8, 10, 20, 30, 40, 50, and 60 individuals. The results showed that the number of alleles and the Polymorphism Information Content (*PIC*) were different among the four accessions. Cluster analysis by Unweighted Pair Group Method with Arithmetic Mean (UPGMA) and STRUCTURE placed the 240 individuals into four distinct clusters. The Expected Heterozygosity (*H_E_*) and *PIC* increased along with an increase in sampling size from 1 to 10 plants but did not change significantly when the sample sizes exceeded 10 individuals. At least 90% of the genetic variation in the four germplasms was represented when the sample size was 10. Finally, we concluded that 10 individuals could effectively represent the genetic diversity of one vetch population based on the SCoT markers. This study provides theoretical support for genetic diversity, cultivar identification, evolution, and marker-assisted selection breeding in common vetch.

## 1. Introduction

Common vetch (*Vicia sativa* L. subsp. *sativa* L.), known as an important self-pollinating forage legume [1,2], is one of the most commonly grown cover crops in the world [3] and is used as pasture, hay and silage [4]. Common vetch also has the ability to grow across a complex range of climate and soil conditions [5,6,7]. In addition, common vetch can fit well into cereal rotations (intercrops) to reduce the incidence of diseases in succeeding non-legume crops [8]. Due to the advantages of its economic value and ecological protection, common vetch is widely cultivated in many parts of the world, including Turkey, China, eastern Asia, India and the USA [1,9,10]. Although common vetch has a high research value, there is currently no scientific sampling size for this species, which has hindered species-related research to some extent. In order to obtain relatively high genetic diversity from a population and select a few individuals in a given region for the efficient conservation of common vetch, an appropriate sampling size should be considered. The method of collecting many seeds from one or a few individuals will result in the loss of the majority of genetic diversity in a population. If more individuals are included in a sample, the sampling size will encompass a high level of diversity, but an exceedingly large sample size from a population will increase the workload for the maintenance and management of the germplasm, particularly when the seeds have to be reproduced in the germplasm storage. To ensure efficient, economical, and maximal retention of the genetic diversity of each vetch population during sampling, it is necessary to have an optimal sampling size to capture the diversity with a manageable number of individuals in one population. To achieve this sampling size, detailed analysis of population genetic diversity and comparison of the genetic constitution of common vetch should be implemented.

With the development of molecular marker technology, a variety of molecular markers have been used to evaluate genetic diversity in crops. Examples include the amplified fragment length polymorphism (AFLP) in *Taraxacum koksaghyz* Rodin [11], single nucleotide polymorphism (SNP) in *Miscanthus* [12], restriction fragment length polymorphism (RFLP) in rubber tree (*Hevea* spp.) [13], and simple sequence repeats (SSR) in Alfalfa (*Medicago sativa*) [14] and in Siberian wild rye (*Elymus sibiricus*) [15]. The start codon targeted (SCoT) polymorphism is a new marker system, and it was developed based on the short-conserved region flanking the adenine–thymine–guanine (ATG) start codon in plant genes [16,17]. SCoT is similar to the random amplified polymorphic DNA (RAPD) and inter simple sequence repeat (ISSR) because a single primer is used as forward and reverse [18]. SCoT marker has been successfully applied in mango (*Mangifera indica* L.), grape (*Vitis vinifera* L.), peanut (*Arachis hypogaea* L.), Siberian wild rye (*Elymus sibiricus* L.), and chickpea (*Cicer arietinum* L.) [18,19,20,21,22].

To determine the appropriate number of individuals that should be sampled to represent reasonable genetic diversity in a population, we designed an experiment to study the genetic variation patterns of different populations of common vetch with SCoT markers. The optimized sampling size could provide useful information for studies of genetic diversity, cultivar identification, evolution, and marker-assisted selection breeding in common vetch.

## 2. Results

### 2.1. The Polymorphism and Genetic Diversity Analysis of SCoT Markers

**Figure 1:**
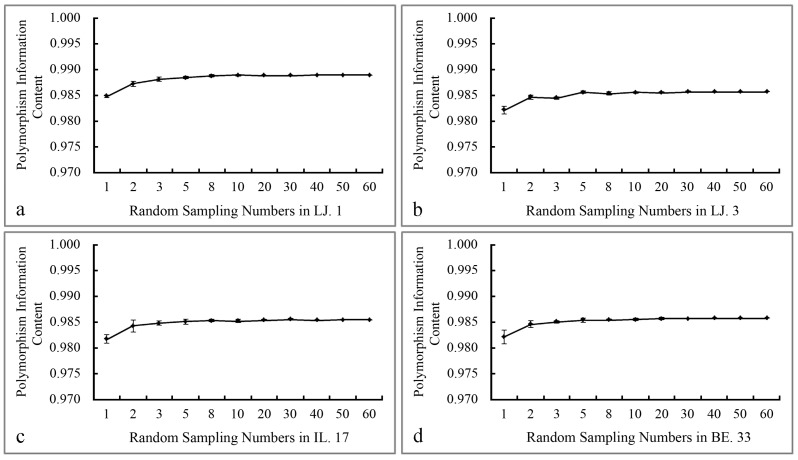
Polymorphism Information Content of the four accessions at different sampling sizes. (**a**) LJ. 1; (**b**) LJ. 3; (**c**) IL. 17; (**d**) BE. 33.

Alleles and genetic diversity parameters are important indicators of the genetic characteristics of the species. In this study, we calculated the genetic diversity parameters in four vetch accessions (Table 1). We observed that the five SCoT primers amplified 122 alleles with an average of 24.4 alleles per locus in the four vetch accessions. Furthermore, the Percentage of Polymorphic Bands (PPB) of five primers were all 100, indicating that the five primers had very high specificity in these four populations. The primers that we selected had high *PIC* (Figure 1) and high *H_E_* (Table 2).

Across the individuals that were in different sampling sizes, when the sample size was less than 10 individuals, the *H_E_* values gradually increased, but at sample sizes greater than 10 individuals, the changes in the *H_E_* values tended to be stable (Table 2). The *PIC* values increased with the increase in sample sizes up to 10 individuals, and reached a plateau in sample sizes greater than 10 individuals (Figure 1). The Number of allele (*Na*) and the Reserve Percentage (RP) of the four accessions increased along with the sampling size increase from 1 to 10 individuals but did not change obviously when the sample size exceeded 10 individuals. The RP value of the four germplasms in different sampling sizes represented more than 90% of the genetic variation in the total population when the sample size was 10 (Table 2). The Fixation index (FST) values are an important index to determine the degree of genetic differentiation of germplasm. The FST values were ranged from 0.25 to 0.45. With the increase of sampling size up to 10, the value of FST gradually increased (Table 3). The Shannon information index (*I*) of four accessions ranged from 0.67 to 0.69 (Table 3), indicating the high biodiversity within the accessions. The Resolving Power (Rp) of the five SCoT primers ranged from 2.67 to 13.90; the values were inferred based on the ability of the primer to differentiate between genotypes (Table 4).

### 2.2. Cluster and Population Structure Analysis

**Figure 2:**
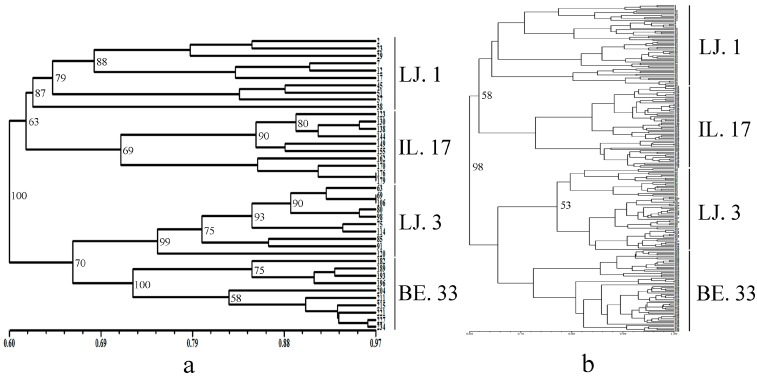
The Unweighted Pair Group Method with Arithmetic Mean (UPGMA) dendrogram of common vetch based on Jaccard’s genetic similarity; only bootstrap values higher than 50% are presented. (**a**) Forty individuals of four germplasm; (**b**) 240 individuals of four germplasm and the genetic distance of start codon targeted (SCoT) markers.

**Figure 3:**
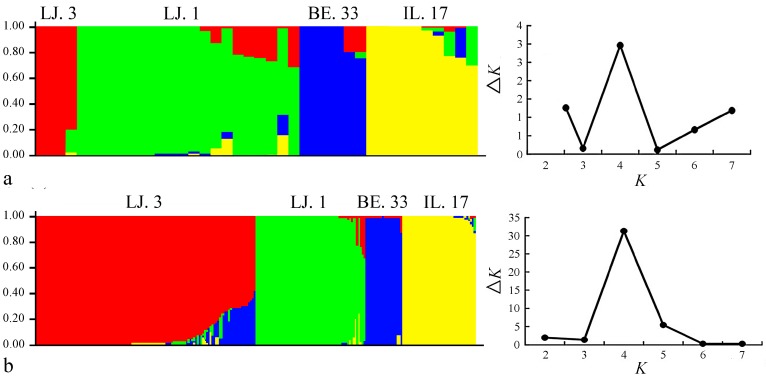
STRUCTURE analysis of the genetic structure of the four vetch accessions. The maximum delta *K* (Δ*K*) values were calculated to determine the topmost level of structure for *K* ranging from 2 to 8 (each with 10 independent runs). Here, *K* was four and four clusters. One color represented one cluster. The red: LJ. 3; The green: LJ. 1; The blue: BE. 33; The yellow: IL. 17. (**a**) The genetic structure of 10 individuals for each of the four accessions as inferred by STRUCTURE with the SCoT markers data set; (**b**) The genetic structure of 60 individuals for each of the four accessions as inferred by STRUCTURE with the SCoT markers data set.

We used the Unweighted Pair Group Method with Arithmetic Mean (UPGMA) and STRUCTURE 2.3.4 software to investigate the relationships between the different sampling levels. According to the UPGMA dendrogram (Figure 2), based on Jaccard’s genetic similarity, only bootstrap values higher than 50% are presented. If the bootstrap value was less than 50%, it cannot sufficiently provide the meaning and polymorphic phylogeny of the accessions [23,24]. The clustering result showed that the sampling level of 10 individuals was consistent with that of 60 individuals. The individuals of the different sampling sizes were assigned to four linkage clusters. The 240 individuals from four different populations were clustered together, and the populations from one location, such as LJ. 1 and LJ. 3, were not clustered together. This finding confirmed the genetic diversity of common vetch from another perspective. The STRUCTURE 2.3.4 software was used to verify the feasibility of the proposed random sampling results. It was run for *K* = 2–8 based on the distribution of the five SCoT primers among the individuals at different sampling levels. Each color represents one cluster. In Figure 3, we can see that STRUCTURE inferred four clusters. In addition, based on the maximum likelihood and delta *K* (Δ*K*) values, we found that whether the sampling level was 10 or 60, the number of optimum groups was four (Figure 3). The two types of software demonstrated that the number of the clusters was consistent with the number of randomly selected germplasms and that a random sampling level at 10 individuals can represent most of the genetic diversity obtained with 60 individuals.

## 3. Discussion

Genetic diversity is one of the most important indicators of biodiversity and is the basic material for the survival and reproduction of species. The study of the genetic diversity of different accessions not only clearly elucidated the genetic structure and characteristics of their populations but also provided technical support for the utilization and protection of their germplasm resources, which has far-reaching implications for endangered or complex species [16].

Common vetch is an annual and strictly self-pollinating forage legume. According to the biological principle on population genetics of species with different breeding systems, comparatively rich genetic diversity should be expected within this species, which may be caused by the trait of a strict closed pollination for the common vetch [25]. Presently, molecular genetic diversity of common vetch is mainly focused on EST-SSR markers and AFLP [2,3,4,5,6]. SCoT markers were firstly introduced in the common vetch, and showed a relatively higher polymorphic than EST-SSR markers [2,4,5].

The proposed statistical models to obtain a general estimation of the number of samples that should be collected to represent a population [25,26] determined that if the population is a Mendelian population, then the appropriate number of individuals is 30 for a plant species to properly represent an ideal population [26]. For wild soybean (*Glycine soja*) populations, researchers studied the germplasms in several areas and determined that they only needed to sample 27 to 52 individuals for the representation of most of the genetic information of the accessions [27]. However, for the *Agropyron* Gaertn. species, the genetic diversity of different germplasms and individuals were studied by random sampling, and the researchers concluded that at least 12 individuals can represent the majority of the genetic diversity of the species [28]. This finding indicates that there are differences in the genetic information of different species and that the corresponding sampling sizes are also significantly different. In this study, we used SCoT markers to study common vetch. Compared with other molecular markers such as SSR, AFLP, and SSAP, SCoT markers have the advantages of simple operation, low cost and abundant polymorphisms, and they are more conducive to molecular-assisted breeding. Based on the DNA products amplified from the five selected SCoT primers, the experiment found high genetic variation in the common vetch population, with 100% of polymorphic loci. The high values of polymorphic loci were also found in orchardgrass (*Dactylis glomerata* L.) and *Trichoderma koningii* using SCoT markers [29,30]. Furthermore, the FST, *H_E_*, *PIC* and *I* values of four accessions also showed the high level of genetic diversity for common vetch accessions.

In the present study, alleles and the RP values were used as the criterion for estimating the genetic diversity of the four common vetch populations, which include both cultivated varieties and wild accessions. As indicated in Table 2, the increase in genetic diversity shows a strong correlation with the increasing number of randomly collected samples with 1, 2, 3, 5, 8, 10, 20, 30, 40, 50, and 60 individuals. The increase in genetic diversity is dramatic with an increase in individuals of up to 10, and the increase in genetic diversity becomes slow and rapidly reaches a plateau when more than 10 individuals are included in the sample set. We concluded that a set of 10 randomly collected individuals can represent the majority of genetic diversity in the population, of which the RP value of alleles is over 90%. In general, more than 90% of RP values of the alleles can represent most of the germplasm genetic information. We concluded that the random sampling size of 10 individuals could highly represent the genetic information of common vetch populations.

Compared with the genetic diversity of the wild accessions IL. 17 and BE. 33, the genetic diversity of the cultivated varieties LJ. 1 and LJ. 3 showed higher values at the same sampling level, which are reflected in the indices of *H_E_*, *PIC*, *Na*, and RP (Table 2). We concluded that LJ. 1 and LJ. 3, as cultivated varieties, underwent individual selection during breeding activities and had higher levels of homozygosity among individuals.

The development of a core collection for a plant would provide a subset of accessions that represented the most diversity of the entire collection. As a polymorphic molecular marker technology, SCoT would be useful for evaluating the genetic diversity in the development of the core collection in the vetch. To screen a core collection from thousands of vetch accessions, 10 individuals per accession would require more time and labor. Based on all statistical indexes in different sampling levels (Figure 1), we suggest that five individuals per accession would be optimal. At this sampling level, the RP values of alleles exceeded 80% (Table 2).

## 4. Materials and Methods

### 4.1. Plant Materials and DNA Extraction

In total, 240 individuals of four common vetch accessions were used in this study (Table 1). The seeds of four accessions were supplied by the National Plant Germplasm System (NPGS, America) and Lanzhou University. The seeds of the four accessions were sown in the Yuzhong Experimental Station of Lanzhou University (N 35°57′, E 104°09′), Lanzhou, China. The annual precipitation is 400 mm, and the mean annual temperature is 6.7 °C (from the year 2013 to 2015) [31]. All accessions in a total of 240 individuals were used for the genetic diversity analysis. Sixty individuals of each accession were sampled for the extraction of DNA. Leaf samples were obtained from young plants, and a revised cetyltrimethylammonium ammonium bromide (CTAB) method [32] was used for the extraction of DNA. The DNA quality was determined using a Nanodrop spectrophotometer (NanoDrop Products, Wilmington, DE, USA), and the DNA concentration was determined using 1.4% agarose gel electrophoresis. Eventually, the extraction DNA were diluted to 25 ng/μL and stored at −20 °C for further PCR amplification.

### 4.2. PCR Amplification

In this experiment, a total of 25 SCoT primers were screened by using agarose gel electrophoresis (Table 5) [17,19]. After screening and optimization, we selected five primers with higher polymorphisms and clear bands (Table 6).

The PCR reactions were all conducted in volumes of 10 μL containing 2.0 μL 25 ng/μL DNA, 5.0 μL 2× Power Taq PCR Master Mix (Bioteke, Beijing, China), 1.0 μL of each primer and 2.0 μL double-distilled water. The PCR programs were set at 94 °C for 4 min, followed by 35 cycles of 1 min at 94 °C, 1 min at 50 °C and 2 min at 72 °C, with a final extension at 72 °C for 7 min. After PCR amplification, fragments were separated in 1.4% agarose gel containing 0.14 μg/mL of Goldview through electrophoresis in 1× TBE buffer solution at 129 V for 2 h 15 min. DNA fragments were visualized under the ultravlolet (UV) light Gel Doc (TM) XR System (Bio-Rad, Hercules, CA, USA), and photo documentation was obtained for each gel.

### 4.3. Data Analysis

The amplified bands were scored as absent (0) or present (1), and only reproducible bands were considered. To evaluate the genetic diversity within the germplasm, the indexes of Expected Heterozygosity (*H_E_*), Polymorphic Information Content (*PIC*), and the observed number of alleles (*Na*) were calculated as previously described [4,33]. Using the number of alleles observed in a sample to estimate the number of different alleles at a single locus in a population [34]. The Reserve Percentage (RP), which is an important indicator of the proportion of alleles remaining in the sample population was calculated [35], and the Analysis of Molecular Variance (AMOVA) was used to partition the total genetic variation among species, among populations within species and within populations via AMOVA Version 1.55 [36]. A cluster analysis was performed to generate a dendrogram; the dendrogram which was constructed by Jaccard’s genetic similarity matrix to display accession relationships using the UPGMA and Nei’s unbiased genetic distance with the help of SAHN clustering via the NTSYS-pc.V.2.1 software package [37], and the bootstrap values were calculated by free tree + tree view (version 1.6.6 for Windows) [38]. A model-based approach implemented in the software program STRUCTURE 2.3.4 was used to subdivide the individuals into different subgroups [39]. We used the ad hoc measure ∆*K* [40] to estimate the number of groups. Under the admixture model of the STRUCTURE software, a burn-in period of 10,000 iterations and a run of 100,000 replications of Markov Chain Monte Carlo after burn-in were performed. The membership of each genotype was tested for the range of genetic clusters from *K* = 2 to *K* = 8 (each with 10 independent runs). The FST values of the alleles of different sampling sizes were also calculated by the software ARLEQUIN version 3.11 [41]. The Rp value of the primer in each accession was measured in accordance with Rp = ΣI_b_ [42]. The Shannon information index (*I*) values between four accessions were analyzed using POPGENE 32 Version 1.31 [43].

## 5. Conclusions

In this study, four different germplasms including 240 individuals were used for the determination of a sampling size for common vetch. The results showed differing degrees of genetic variation on the various sampling levels. Based on the results, we concluded that a sample of 10 individuals is optimal for future studies of common vetch, as this sampling size can represent over 90% of the genetic diversity of the population. This sampling size could provide technical support for molecular breeding and the protection of germplasm of common vetch.

## Figures and Tables

**Table 1:** Names of common vetch accessions and their origins.

Accessions	Type of Accessions	Sample Code	Origin of Germplasm
LJ. 1	Cultivated variety, Lanjian No. 1	1–60	China
LJ. 3	Cultivated variety, Lanjian No. 3	61–120	China
IL. 17	Wild accession, No. 17	121–180	Israel
BE. 33	Wild accession, No. 33	181–240	Belgium

**Table 2:** Expected heterozygosity (*H_E_*), the observed number of alleles (*Na*) and the Reserve Percentage (RP) at different sampling sizes of the four accessions.

Sampling No. (Sampling Size)	LJ. 1			LJ. 3			IL. 17			BE. 33		
	*Na*	RP	*H_E_*	*Na*	RP	*H_E_*	*Na*	RP	*H_E_*	*Na*	RP	*H_E_*
1 (1)	67	58%	0.9851	59	56%	0.9823	56	57%	0.9822	58	55%	0.9825
2 (2)	87	75%	0.9874	72	69%	0.9847	71	72%	0.9846	72	69%	0.9849
3 (3)	97	84%	0.9882	74	70%	0.9845	79	80%	0.9851	79	75%	0.9853
4 (5)	104	89%	0.9886	84	80%	0.9856	87	88%	0.9854	85	81%	0.9857
5 (8)	108	93%	0.9889	88	84%	0.9854	88	89%	0.9856	91	87%	0.9857
6 (10)	112	97%	0.9890	95	90%	0.9856	91	92%	0.9856	94	90%	0.9858
7 (20)	113	97%	0.9889	96	91%	0.9855	95	96%	0.9857	98	93%	0.9860
8 (30)	114	98%	0.9890	100	95%	0.9857	97	98%	0.9858	100	95%	0.9859
9 (40)	115	99%	0.9890	102	97%	0.9857	98	99%	0.9857	104	99%	0.9860
10 (50)	116	100%	0.9890	103	98%	0.9857	99	100%	0.9858	104	99%	0.9860
11 (60)	116	100%	0.9890	105	100%	0.9857	99	100%	0.9858	105	100%	0.9860

**Table 3:** The Fixation index values (FST) and the Shannon information index of the four accessions.

Sampling No. (Sampling Size)	LJ. 1	LJ. 3	IL. 17	BE. 33
1 (1)	0.00	0.00	0.00	0.00
2 (2)	0.25	0.31	0.33	0.34
3 (3)	0.34	0.37	0.36	0.37
4 (5)	0.35	0.40	0.39	0.39
5 (8)	0.39	0.41	0.42	0.42
6 (10)	0.44	0.44	0.44	0.45
7 (20)	0.44	0.43	0.44	0.45
8 (30)	0.43	0.44	0.44	0.44
9 (40)	0.44	0.45	0.45	0.45
10 (50)	0.45	0.45	0.45	0.45
11 (60)	0.45	0.45	0.45	0.45
Shannon information index	0.67	0.68	0.69	0.69

**Table 4:** The Resolving Power (Rp), the Expected Heterozygosity (*H_E_*) and Polymorphism Information Content (*PIC*) of each primer in each accession.

Primer	LJ. 1			LJ. 3			IL. 17			BE. 33		
	Rp	*H_E_*	*PIC*	Rp	*H_E_*	*PIC*	Rp	*H_E_*	*PIC*	Rp	*H_E_*	*PIC*
Scot28	8.53	0.9442	0.9413	5.90	0.9282	0.9235	3.07	0.8834	0.8720	6.00	0.9114	0.9046
Scot35	13.67	0.9413	0.9381	6.40	0.9216	0.9160	10.73	0.9344	0.9305	11.10	0.9345	0.9306
Scot36	13.90	0.9518	0.9496	7.50	0.9194	0.9138	4.20	0.9374	0.9337	7.43	0.9216	0.9161
Scot37	9.17	0.9408	0.9376	4.33	0.9248	0.9197	9.10	0.9275	0.9227	6.10	0.9309	0.9266
Scot38	8.63	0.9511	0.9488	5.87	0.9442	0.9412	6.03	0.9445	0.9416	2.67	0.9492	0.9467

**Table 5:** Information for the 25 SCoT primers used in screening in materials.

Primer	Primer Sequence (5′-3′)	Primer	Primer Sequence (5′-3′)
Scot12	ACGACATGGCGACCAACG	Scot41	CAATGGCTACCACTGACA
Scot13	ACGACATGGCGACCATCG	Scot42	CAATGGCTACCATTAGCG
Scot14	ACGACATGGCGACCACGC	Scot43	CAATGGCTACCACCGCAG
Scot15	ACGACATGGCGACCGCGA	Scot44	CAATGGCTACCATTAGCC
Scot16	ACCATGGCTACCACCGAC	Scot45	ACAATGGCTACCACTGAC
Scot23	CACCATGGCTACCACCAG	Scot46	ACAATGGCTACCACTGAG
Scot28	CCATGGCTACCACCGCCA	Scot47	ACAATGGCTACCACTGCC
Scot35	CATGGCTACCACCGGCCC	Scot48	ACAATGGCTACCACTGGC
Scot36	GCAACAATGGCTACCACC	Scot49	ACAATGGCTACCACTGCG
Scot37	CAATGGCTACCACTAGCC	Scot50	ACAATGGCTACCACTGGG
Scot38	CAATGGCTACCACTAACG	Scot51	ACAATGGCTACCACTGTC
Scot39	CAATGGCTACCACTAGCG	Scot52	ACAATGGCTACCACTGCA
Scot40	CAATGGCTACCACTACAG		

**Table 6:** Information for the five SCoT primers used in the genetic diversity analysis in common vetch.

Primer	Tm/°C	PPB	*Na*	*H_E_*	*PIC*	Range of Band Size (bp)
Scot28	61.9	100%	25	0.9374	0.9339	200–2100
Scot35	64.1	100%	21	0.9429	0.9400	200–1900
Scot36	57.3	100%	29	0.9540	0.9520	240–2200
Scot37	61.9	100%	23	0.9444	0.9415	250–2400
Scot38	61.9	100%	24	0.9518	0.9496	220–1800
Average		100%	24.4	0.9461	0.9434	

Note: PPB: Percentage of Polymorphic Bands; *Na*: Observed number of alleles; *H_E_*: Expected Heterozygosity; *PIC*: Polymorphic Information Content.
